# "Lock-and-Advance" Technique: A Triple Co-axial System With a Steerable Microcatheter for Retrograde Embolization of a Ruptured Splenic Artery Aneurysm

**DOI:** 10.7759/cureus.106114

**Published:** 2026-03-30

**Authors:** Yoshihiro Kubota, Seiki Shimoda, Satoru Mikami, Naoki Kogayo, Takashi Uno

**Affiliations:** 1 Department of Radiology/Diagnostic Radiology, Chiba University Hospital, Chiba, JPN; 2 Department of Radiology, Chiba University Hospital, Chiba, JPN; 3 Department of Radiology, Chiba University Graduate School of Medicine, Chiba, JPN; 4 Department of Radiology, University of Chiba, Chiba, JPN

**Keywords:** coil-embolization, lock-and-advance technique, splenic artery aneurysm rupture, steerable microcatheter, trans-collateral approach

## Abstract

A ruptured splenic artery aneurysm (SAA) is a life-threatening condition requiring urgent endovascular isolation. However, antegrade access can be challenging due to severe tortuosity or anatomical changes post-gastrectomy. An 80-year-old woman presented with a ruptured 45 mm pseudoaneurysm originating from a SAA five days after femoral fracture surgery. Initial antegrade access failed because of a narrowed and tortuous entry to the distal artery. We performed a retrograde trans-collateral approach via the transverse pancreatic artery. A triple co-axial system was used, featuring a 2.9-Fr steerable microcatheter (Leonis Mova HF; Sumitomo Bakelite, Tokyo, Japan), a 1.9-Fr microcatheter (Carnelian MARVEL; Tokai Medical Products, Aichi, Japan), and a 0.010-inch guidewire (CHIKAI X010; ASAHI INTECC, Tokyo, Japan). By locking the steerable microcatheter at curvatures to enhance backup support, the inner system successfully navigated the tortuous collaterals to achieve distal embolization. The "lock-and-advance" technique using a steerable microcatheter may be a feasible and useful option for navigating delicate collateral pathways in emergency settings.

## Introduction

Splenic artery aneurysm (SAA) rupture is a critical emergency with high mortality rates [1]. The standard treatment is endovascular isolation, which involves embolizing both the proximal and distal segments of the aneurysm to prevent backflow [2]. Anatomical variations, such as those following gastrectomy, can lead to the development of specific collateral pathways like the transverse pancreatic artery (TPA) [3,4]. When antegrade access to the distal artery is blocked by severe tortuosity or narrowing, a trans-collateral approach becomes necessary [3]. However, navigating these small, winding vessels carries a risk of injury and often lacks the mechanical support needed for catheter advancement. While steerable microcatheters are established in other fields like neuro-intervention and hepatic interventions, their specific application as a 'locking dynamic anchor' to overcome this lack of support in visceral collaterals remains underreported [5-7]. We describe a successful case of retrograde SAA embolization using a "lock-and-advance" technique with a steerable microcatheter (Leonis Mova HF; Sumitomo Bakelite, Tokyo, Japan).

## Case presentation

**Figure 1:**
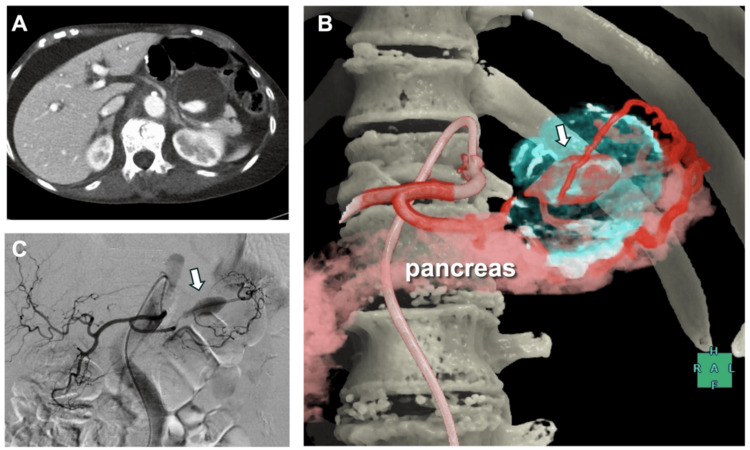
Imaging findings of the pancreatic tail pseudoaneurysm (A) Contrast-enhanced computed tomography (CT) showing a mass in the tail of the pancreas, consistent with a hematoma, with evidence of active contrast extravasation within the lesion. (B, C) CT-angiography (CTA) and digital subtraction angiography (DSA) revealing a pseudoaneurysm arising from the main trunk of the splenic artery (white arrow). Note the development of collateral circulation from the dorsal pancreatic artery to the splenic hilum through the transverse pancreatic artery.

An 80-year-old woman underwent open reduction and internal fixation for a left femoral trochanteric fracture. Five days postoperatively, she developed oliguria, abdominal distension, and tenderness. Contrast-enhanced CT revealed a 45 mm pseudoaneurysm resulting from a ruptured SAA with active extravasation (Figure 1).

**Figure 2:**
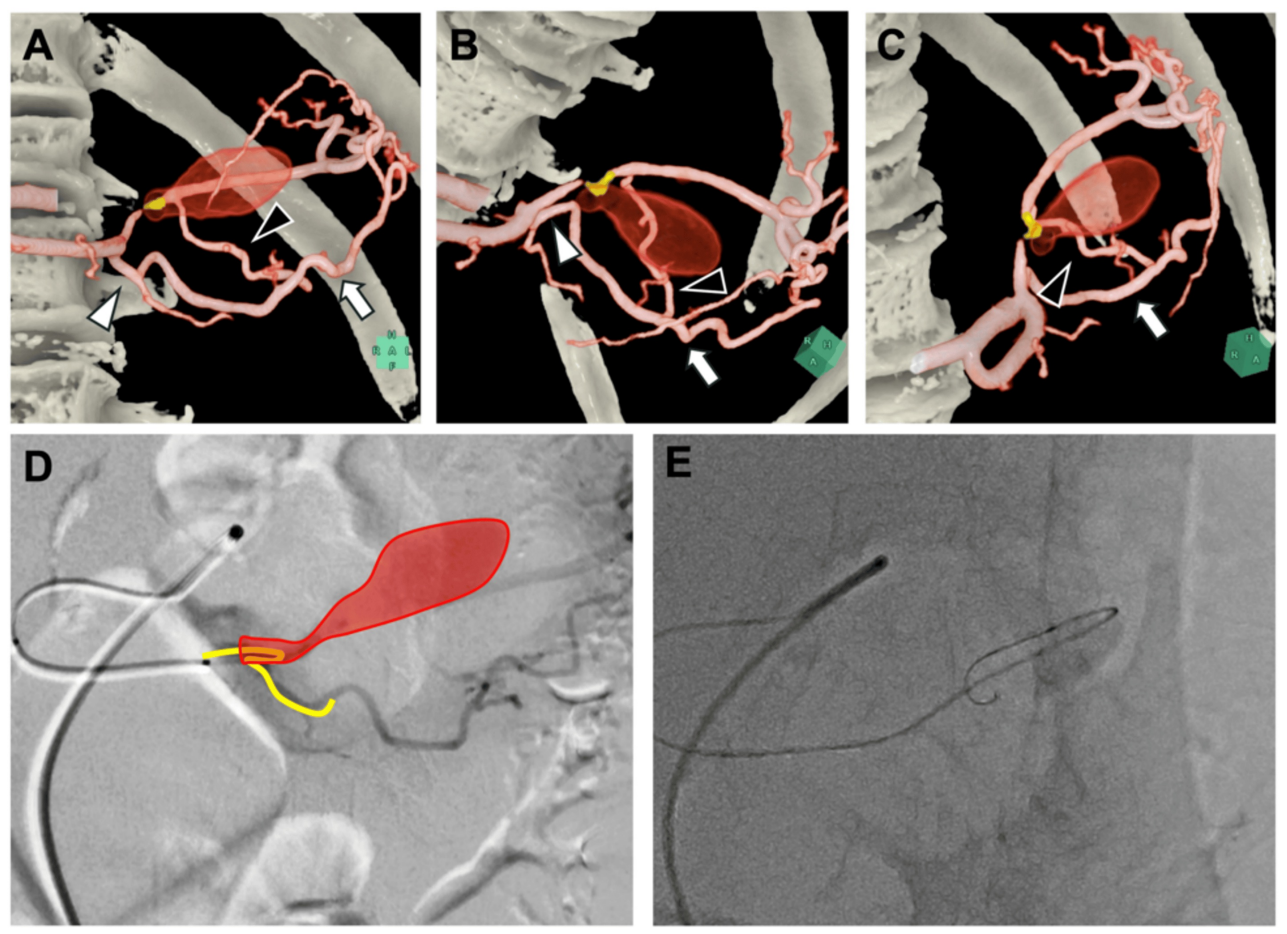
Trans-arterial computed tomography angiography (CTA) and digital subtraction angiography (DSA) (A-C) Multi-projection CTA images demonstrate the narrow, tortuous distal splenic artery. The dorsal pancreatic artery (white arrowhead), TPA (white arrow), and artery pancreatica magna are identified. (D) DSA with schematic overlay highlighting marked tortuosity distal to the aneurysm. (E) Failed selective catheterization due to the aneurysm’s size and distal anatomy. TPA: Transverse pancreatic artery

The patient was transferred for emergency intervention. Upon arrival at our hospital, her hemodynamic status was stable, with a blood pressure of 125/52 mmHg, a heart rate of 53 beats per minute, and a serum lactate level of 1.1 mmol/L (reference range: 0.36-0.75 mmol/L). The procedure began with a right femoral approach under local anesthesia and conscious sedation. Celiac artery angiography showed the SAA and prominent collateral flow to the spleen via the TPA and greater pancreatic artery. Due to a prior gastrectomy, the left gastric and gastroepiploic arteries were absent. An initial antegrade attempt using a 2.4/2.6-Fr microcatheter and a 0.014-inch guidewire was unsuccessful due to the narrow and tortuous distal entry point (Figure 2).

**Figure 3:**
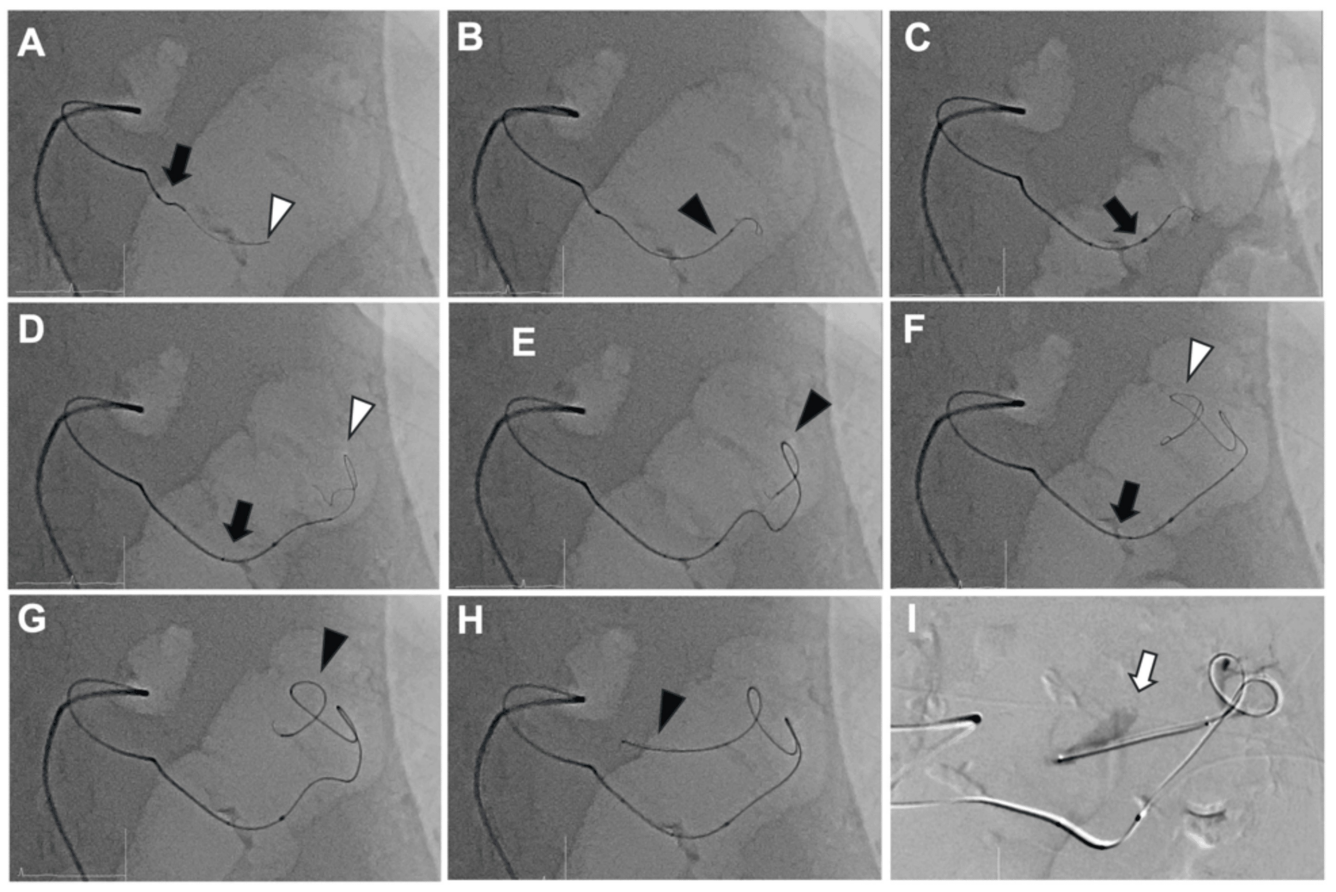
Step-by-step fluoroscopic images of the "lock-and-advance" technique via a trans-collateral approach The symbols indicate the following: black arrows for the mother catheter (Leonis Mova HF), white arrowheads for the micro-guidewire, and black arrowheads for the child microcatheter (Carnelian Marvel). (A, B) The mother catheter is locked at a vessel curvature to provide backup support while the guidewire and child catheter are advanced distally. (C) The mother catheter is then unlocked and advanced over the child system. (D-G) Through repeated cycles of locking the mother catheter and advancing the inner system, stable navigation is maintained through the tortuous segments. (H, I) The system successfully reaches the target segment distal to the aneurysm (white arrow).

We shifted to a trans-collateral retrograde approach via the TPA using a triple co-axial system consisting of a 2.9-Fr Leonis Mova HF mother catheter, a 1.9-Fr Carnelian MARVEL (Tokai Medical Products, Aichi, Japan) child microcatheter, and a 0.010-inch guidewire (CHIKAI X010; ASAHI INTECC, Tokyo, Japan). To navigate the TPA, we employed a repetitive "lock-and-advance" technique. First, the steerable tip of the Leonis Mova HF was shaped to match the vessel's curvature and locked to provide a rigid backup platform. This allowed the 0.010-inch guidewire and the child microcatheter to advance through the tortuous segments without the mother catheter being pushed back (kick-back). Once the inner system was distal, the mother catheter was unlocked, advanced over the child, and re-locked at the next bend. This iterative process allowed us to safely reach the distal splenic artery (Figure 3).

**Figure 4:**
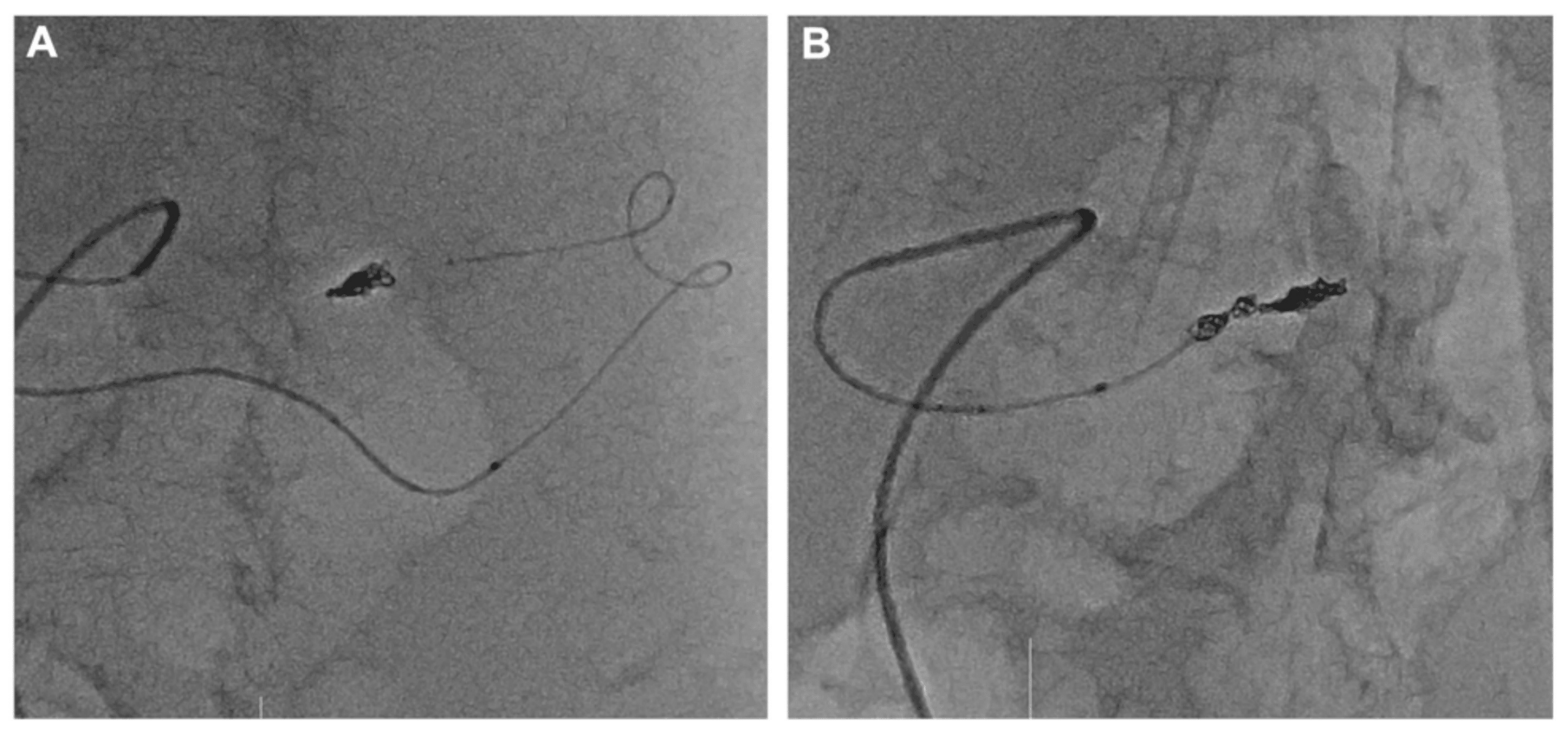
Isolation of the pseudoaneurysm with coil embolization (A) Retrograde coil embolization of the splenic artery immediately distal to the pseudoaneurysm was performed via the trans-collateral approach using four Azur Soft 3D coils. (B) The splenic artery proximal to the pseudoaneurysm was subsequently embolized with two Azur Soft 3D coils and one Avenir coil to achieve complete isolation of the lesion.

Distal embolization was performed using three AZUR Soft3D coils (1 mm x 3 cm, 1.5 mm x 4 cm, and 1 mm x 3 cm; Terumo, Tokyo, Japan). The system was then withdrawn to the proximal splenic artery, where further isolation was completed using an AZUR Soft3D coil (2 mm x 6 cm), an Avenir Helical coil (4 mm x 20 cm; Wallaby Medical, Laguna Hills, California, US), and an additional AZUR Soft3D coil (1.5 mm x 4 cm) (Figure 4).

**Figure 5:**
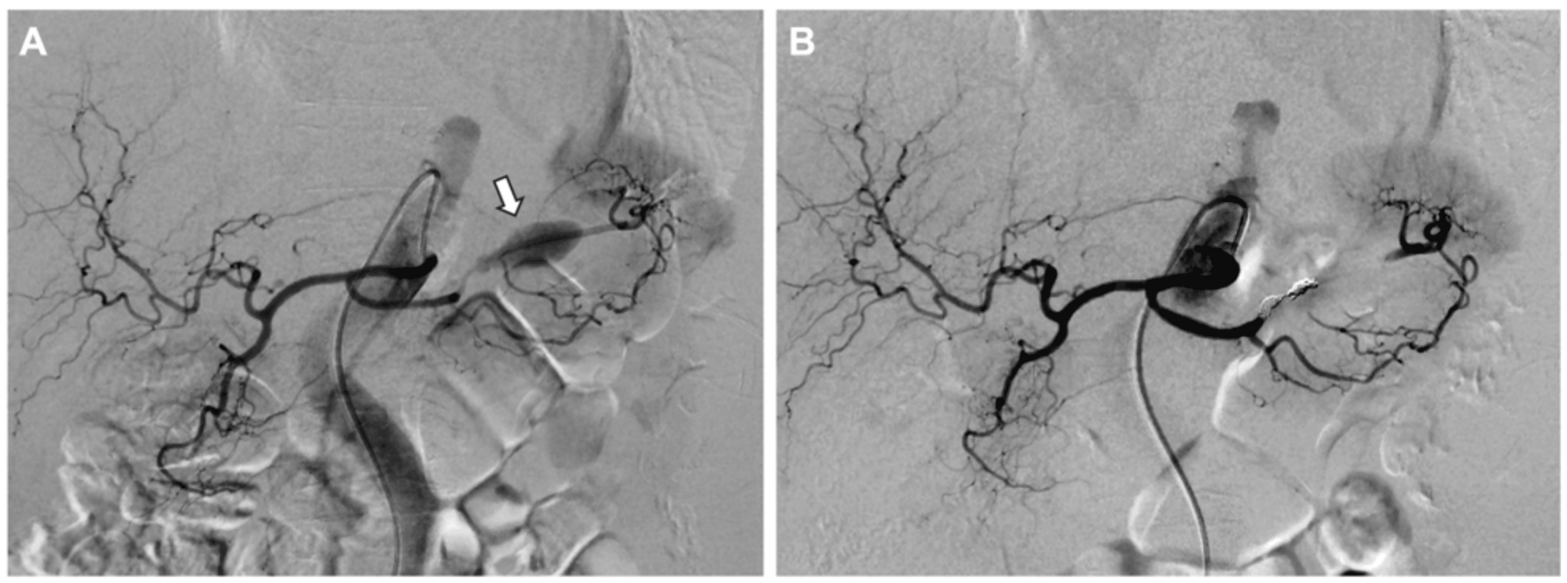
Pre- and post-embolization angiographic findings (A) Pre-embolization angiography shows the patent splenic artery aneurysm with contrast opacification. (B) Final angiography demonstrates the complete disappearance of contrast opacification within the pseudoaneurysm. No evidence of vascular injury is observed in the collateral pathways. Splenic perfusion is well-preserved through these collateral vessels.

Final angiography confirmed the exclusion of the SAA while preserving splenic perfusion through the TPA (Figure 5).

The total procedure time was 115 minutes, the fluoroscopy time was 37.7 minutes, and the total volume of iodinated contrast media used was 75 ml. The patient remained stable and was transferred back to the referring hospital two days after the procedure for continued postoperative recovery.

## Discussion

Steerable microcatheters as a dynamic anchor

The technical highlight of this case is the use of a steerable microcatheter as a "dynamic anchor". In trans-collateral interventions, the primary challenge is the lack of backup support in small-caliber vessels [3]. Steerable microcatheters have recently demonstrated high success rates in navigating complex vascular anatomy by allowing real-time tip adjustment [5]. While these devices are well-established in fields like neuro-intervention and hepatic intervention, their specific application as a "locking dynamic anchor" to provide mechanical support within fragile visceral collaterals remains underreported.

Mechanism and safety of the lock-and-advance technique

By locking the Leonis Mova HF at curvatures, we effectively converted it into a customized support structure that facilitated the advancement of the 0.010-inch system, a mechanism like the "anchor technique" described in neurovascular interventions [6]. Mechanistically, the locking of the microcatheter at a curvature redistributes the forces involved in advancement. When the inner child system is pushed forward, it typically generates an equal and opposite "kick-back" force that tends to dislodge the mother catheter. By locking the steerable tip against the outer curve of the vessel wall, this longitudinal reaction force is converted into a lateral vector applied against the wall. This lateral anchoring effectively neutralizes the kick-back, providing the necessary stability to navigate distal tortuosity without a traditional distal anchor. While this technique is intended to minimize the risk of vessel dissection or vasospasm by reducing the need for forceful advancement, these benefits remain theoretical in the context of this single case. It is important to note that the "lock-and-advance" maneuver carries inherent risks; the lateral force exerted by the locked tip could potentially cause vessel wall injury or focal vasospasm if excessive tension is applied [7].

Anatomical considerations for a trans-collateral approach

Furthermore, in post-gastrectomy patients, preserving collateral pathways like the TPA is vital for maintaining organ perfusion [4]. In the present case, we opted for a retrograde approach to the pseudoaneurysm, navigating from the dorsal pancreatic artery through the transverse pancreatic artery to the splenic hilar arteries. Although an alternative route existed through the transverse pancreatic artery via the artery pancreatica magna to reach the vessel disruption site directly, we believe that an approach via the artery pancreatica magna would also be feasible by employing the "lock-and-advance" technique demonstrated here. The triple co-axial approach, combined with a locking mechanism, provides the necessary stability to facilitate access to distal targets in emergency settings. However, the inherent limitations of a single case report must be considered. The perceived safety benefits require further validation, as the success of the maneuver may depend on specific vessel diameters, operator experience, and the degree of tortuosity. Its reproducibility across different operators and clinical scenarios warrants further investigation.

## Conclusions

The "lock-and-advance" technique, utilizing a triple co-axial system with a steerable microcatheter, provided a technically feasible and useful option for navigating highly tortuous collateral pathways in this case. By leveraging the microcatheter’s locking mechanism within side branches to provide stable backup support, this approach facilitated the precise delivery of embolic materials to distal targets that were otherwise inaccessible via conventional antegrade routes. In the present emergency setting, this maneuver allowed for the successful isolation of the pseudoaneurysm while preserving splenic perfusion. While this technical adaptation represents a promising option for managing complex visceral artery aneurysms, its broader safety profile and reproducibility warrant further investigation to determine its role in clinical practice.
